# Potential Applications of Nanocellulose-Containing Materials in the Biomedical Field

**DOI:** 10.3390/ma10080977

**Published:** 2017-08-21

**Authors:** Nadia Halib, Francesca Perrone, Maja Cemazar, Barbara Dapas, Rossella Farra, Michela Abrami, Gianluca Chiarappa, Giancarlo Forte, Fabrizio Zanconati, Gabriele Pozzato, Luigi Murena, Nicola Fiotti, Romano Lapasin, Laura Cansolino, Gabriele Grassi, Mario Grassi

**Affiliations:** 1 Department of Basic Sciences & Oral Biology, Faculty of Dentistry, Universiti Sains Islam Malaysia, Level 15, Tower B, Persiaran MPAJ, Jalan Pandan Utama, Kuala Lumpur 55100, Malaysia; nadia.halib@usim.edu.my; 2 Department of Life Sciences, Cattinara University Hospital, Trieste University, Strada di Fiume 447, I-34149 Trieste, Italy; fperrone116@gmail.com (F.P.); bdapas@units.it (B.D.); 3 Institute of Oncology Ljubljana, Zaloska 2, SI-1000 Ljubljana, Slovenia; MCemazar@onko-i.si; 4 Department of Engineering and Architecture, University of Trieste, Via Valerio 6/A, I-34127 Trieste, Italy; rfarra@units.it (R.F.); mikystars@hotmail.com (M.A.); gianluca.chiarappa@phd.units.it (G.C.); romano.lapasin@dia.units.it (R.L.); mario.grassi@dia.units.it (M.G.); 5 Center for Translational Medicine, International Clinical Research Center, St. Anne’s University Hospital, Pekarska 53, 656 91 Brno, Czech Republic; giaforte@gmail.com; 6 Surgery and Health Sciences, Department of Medical, Cattinara Hospital, University of Trieste, I-34127 Trieste, Italy; fabrizio.zanconati@asuits.sanita.fvg.it (F.Z.); G.POZZATO@fmc.units.it (G.P.); lmurena@units.it (L.M.); fiotti@units.it (N.F.); 7 Department of Clinico-Surgical Sciences, Experimental Surgery Laboratory, University of Pavia and IRCCS S, Matteo Hospital Pavia, 27100 Pavia, Italy; lauracansolino@libero.it

**Keywords:** cellulose, wound healing, bone-cartilage regeneration, dental application, siRNA, drug-cell delivery

## Abstract

Because of its high biocompatibility, bio-degradability, low-cost and easy availability, cellulose finds application in disparate areas of research. Here we focus our attention on the most recent and attractive potential applications of cellulose in the biomedical field. We first describe the chemical/structural composition of cellulose fibers, the cellulose sources/features and cellulose chemical modifications employed to improve its properties. We then move to the description of cellulose potential applications in biomedicine. In this field, cellulose is most considered in recent research in the form of nano-sized particle, i.e., nanofiber cellulose (NFC) or cellulose nanocrystal (CNC). NFC is obtained from cellulose via chemical and mechanical methods. CNC can be obtained from macroscopic or microscopic forms of cellulose following strong acid hydrolysis. NFC and CNC are used for several reasons including the mechanical properties, the extended surface area and the low toxicity. Here we present some potential applications of nano-sized cellulose in the fields of wound healing, bone-cartilage regeneration, dental application and different human diseases including cancer. To witness the close proximity of nano-sized cellulose to the practical biomedical use, examples of recent clinical trials are also reported. Altogether, the described examples strongly support the enormous application potential of nano-sized cellulose in the biomedical field.

## 1. Introduction

The use of low-cost and renewable materials has attracted more and more interest from the scientific community. Among renewable resources, cellulose represents a very appealing material. Cellulose is the most abundant and renewable biopolymer in nature. It is the main constituent of plant cell walls in wood, cotton, hemp and other plant-based materials and plays an essential role in maintaining plant structural phase. Cellulose is also synthesized by marine animals such as tunicates, algae, fungi [1,2,3] and several bacteria species belonging to the genera of *Acetobacter*, *Rhizobium*, *Agrobacterium* and *Sarcina* [4]. Cellulose is a carbohydrate homopolymer consisting of *β*-d-glucopyranose units joint together by *β*-1,4-glycosidic linkage [5]. Cellulose fibrils are highly insoluble and inelastic but able to provide mechanical support to the tissues where they resides [6]. Since cellulose is of natural origin, the biocompatibility [7] and bio-degradability are other features scientists are looking for in this amazing material.

Because of the above positive properties, cellulose has found application in different research fields such as the biomedical, energy, environmental and water related fields [8] . In this review, we focus on the cellulose employment in the biomedical field. Particularly, we concentrate our attention on the most recent advances obtained with regard to wound healing (Section 5.1), bone-cartilage regeneration (Section 5.2 and Section 5.3), dental application (Section 5.4), human diseases such as cancer (Section 5.5) and finally on most recent clinical trials (Section 5.6). The description of cellulose potential application in the biomedical field is preceded by the presentation of cellulose structure (Section 2), source (Section 3), and the types/chemical modifications of cellulose used in biomedical studies (Section 4).

## 2. Chemical and Structural Composition of Cellulose Fibers

**Figure 1:**
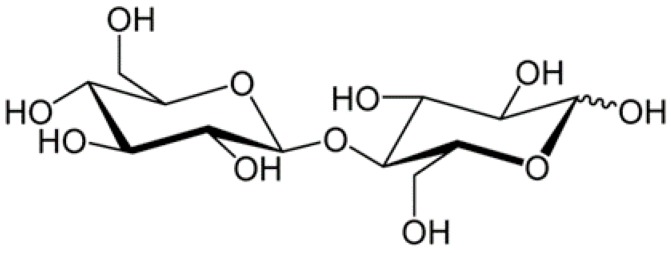
Cellobiose unit (redrawn from [9]).

**Figure 2:**
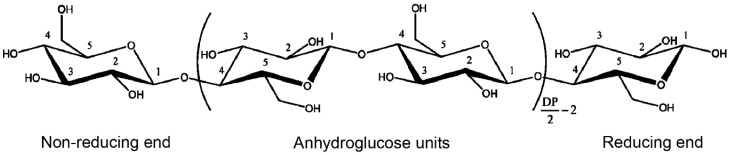
Chemical structure of cellulose (adapted from [11], with permission from © 2010 American Chemical Society).

Cellulose is a high molecular weight natural homopolymer, composed of *α*-1,4-linked anhydro-d-glucose units. The sugar units are linked by combining the H and –OH group together with the elimination of water. Linking two of these sugars produces a disaccharide called cellobiose (Figure 1) [9]. The glucose units are in 6-membered rings, called pyranoses. They are joined by single oxygen atoms (acetal linkages) between the C-1 of one pyranose ring and the C-4 of the next ring. Since the water molecule is lost due to the reaction of an alcohol and a hemiacetal to form an acetal, the glucose units in the cellulose polymer are referred to as anhydroglucose units [10]. The cellulose molecule contains three different kinds of anhydroglucose units, the reducing end with a free hemiacetal (or aldehyde) group at C-1, the nonreducing end with a free hydroxyl at C-4 and the internal rings joined at C-1 and C-4 (Figure 2) [11].

The stereochemistry at carbons 2, 3, 4 and 5 of the glucose molecule are fixed, but in pyranose form, the hydroxyl at C-4 can approach the carbonyl at C-1 from either side, resulting in two different stereo chemistries. If the hydroxyl group at C-1 is on the same side of the ring as the C-6 carbon, it is said to be in the α configuration. In cellulose, the C-1 is positioned oppositely producing β configuration [12]. Functional groups of cellulose β configuration are in equatorial positions, making the cellulose molecular chain to be more or less extended in straight line [10].

**Figure 3:**
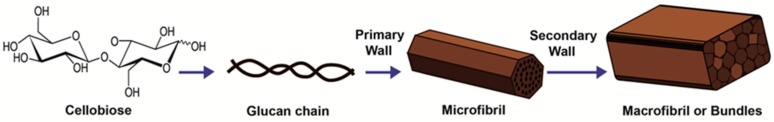
Glucose residues polymerized into individual chain and further assemble into microfibrils and macrofibrils or bundles (redrawn from [9]).

In nature, cellulose does not occur as an isolated individual molecule. Although cellulose is biologically synthesized as individual molecules, they undergo spinning in a hierarchical order at the site of biosynthesis. In the case of primary cell wall of plants, glucose residues are polymerized into individual chains. Later, 36 chains are assembled together to form a microfibril. During synthesis of the plant secondary wall, microfibrils often associate further to form bundles (Figure 3). However, the chain length may varies ranging from as low as 2000 up to 20,000 glucose residues [9].

In the ordered regions, cellulose chains are tightly packed together in crystallites, which are stabilized by a strong and very complex intra- and intermolecular hydrogen-bond network. The hydrogen-bonding network and molecular orientation in cellulose can vary widely, which can give rise to cellulose polymorphs or allomorphs. Six interconvertible polymorphs of cellulose, namely, I, II, III_I_, III_II_, IV_I_, and IV_II_, have been identified. Meanwhile the amorphous regions are distributed as chain dislocations on segments along the elementary fibril where the microfibrils are distorted by internal strain in the fiber and proceed to tilt and twist [13]. Native cellulose has been thought to have cellulose I shape.

## 3. Sources and Properties of Cellulose

Cellulose can be obtained from various sources. The conventional and primary source is from wood of higher plant. Cellulose also origins from agricultural residues such as palm trunk and empty fruit bunch, corncobs, wheat straw, sugarcane bagasse, corn stover, coconut husks, wheat rice and empty fruit bunches [14]. In addition to its plant origins, cellulose fibres are also secreted extracellularly by certain bacteria belonging to the genera *Acetobacter*, *Agrobacterium*, *Alcaligenes*, *Pseudomonas*, *Rhizobium*, or *Sarcina* [15].

Plant cellulose (PC) lies in the xylem tissues that are known as wood where cellulosic microfibrils are embedded in a matrix of hemicellulose (an amorphous non-cellulosic polysaccharides) and lignin [16]. Lignin keeps the water in fibers acting as a stiffener and making the plant stem resistance against gravity forces, wind and provides protection against biological attack. Hemicellulose presence is believed to serve as compatibilizer between cellulose and lignin [17]. Because of the presence of lignin and hemicellulose PC is regarded as less promising for biomedical applications. Indeed, lignin would not be biodegradable under the conditions found in the body and the effects of its prolonged residence in the body would be very hard to predict. Moreover, the harsh chemicals needed to remove lignin and hemicellulose may have deleterious effects in the body due to residual chemicals and their byproducts.

**Figure 4:**
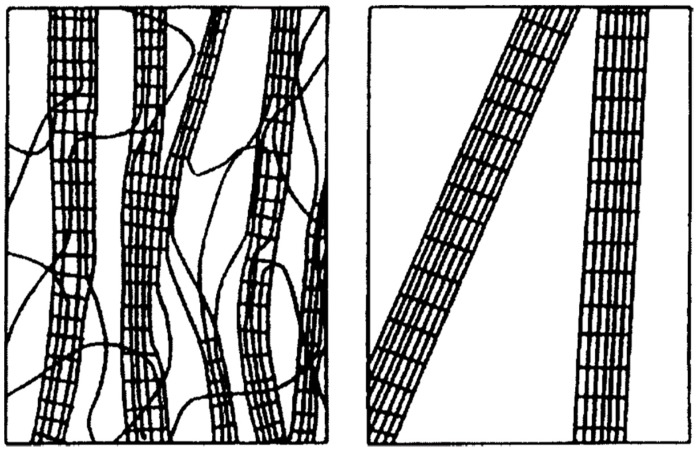
Schematic model of bacterial cellulose (BC) microfibrils (**right**) drawn in comparison with the ‘fringed micelles’ of plant cellulose fibrils (**left**) (adapted from [18], with permission from © 2000 Springer).

In addition to plant, bacteria also synthesize cellulose (bacterial cellulose BC). BC is chemically identical to PC but not in terms of the macromolecular structure and arrangement, hence their properties are different (Figure 4) [18]. BC has been regarded as “generally recognized as safe” by the FDA [19]. The safety of BC stems from the fact that it is free of lignin, hemicellulose structures, does not have any sulfur and it does not need to involve any chemical synthesis and/or treatments. Moreover, purification methods allow obtaining BC with a content of endotoxin falling within the acceptable range indicated by FDA for biomedical applications [19].

**Figure 5:**
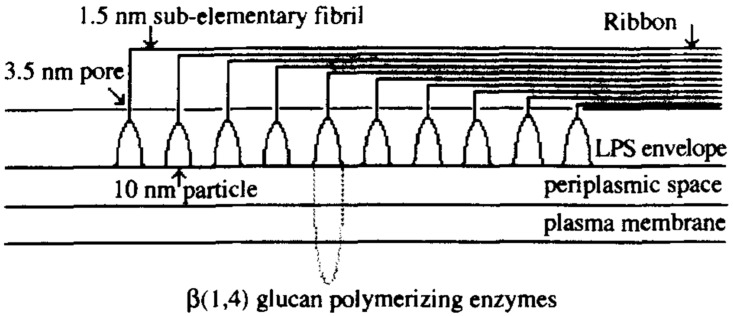
Scheme for the formation of bacterial cellulose (adapted from [4], with permission from © 1998 Elsevier).

BC aggregates to form sub-fibrils, which have the width of approximately 1.5 nm, the thinnest natural occurring fibres. BC sub-fibrils are then crystallized into bundles and latter into ribbons [4] as reported in Figure 5. Several researchers had performed and recorded a few dimensions of BC ribbons. According to Zaar [20] the ribbons were 3–4 nm thick and 70–80 nm long, while Brown et al. [21] reported dimension of 3.2 × 133 nm, whereas Yamanaka et al. [22] noted 4.1 × 117 nm. Other researchers showed that these ribbons (sometimes called fibrils) can reach dimension around 10–100 nm width and 10 μm in length depending on the type of the culture medium and surroundings [6,23,24,25]. Stabilized by extensive hydrogen bonding, these BC ribbons form a dense reticulated structure. BC also differs from plant cellulose by having a higher crystallinity [4]. In addition, BC is produced as a highly hydrated and relatively pure cellulose membrane and therefore no chemical treatments are needed to remove lignin and hemicelluloses, as is the case for plant cellulose [26,27].

With regard to BC synthesis by bacteria, the most important enzyme is cellulose synthase while cyclic diguanylmonophosphate (c-di-GMP) acts as the activator to the cellulose synthase. The production of cellulose from glucose was suggested according to a biochemical pathway by De Wulf et al. [28]. The two most common carbon sources used in bacterial cellulose production are glucose and sucrose. However, many other carbon substrates can be used for cellulose production such as arabitol and mannitol [29,30], glycerol [31] and fructose [32]. In a previous study, Masaoka et al., reported that a low concentration of glucose (less then 15 g/L) could produce up to 0.6 g of cellulose for each gram of glucose per day after 2–4 days of cultivation. For comparison, Oikawa [30] reported that mannitol produced 0.233 g/g/day of cellulose whereas arabitol produced 0.155 g/g/day of cellulose [29]. Finally, beside the above carbon sources, *Acetobacter* can also consume citrate, gluconate, gluconolactone and lactate as its carbon sources [4].

## 4. Isolation and Chemical Modifications of Cellulose

### 4.1. Nanofibrillar Cellulose (NFC)

As mentioned before, native cellulose fibers are built up by mechanically strong and long thin filaments. These are also called microfibrils and consist of alternating crystalline and non-crystalline domains. From microfibrils it is possible to obtain nanofiber cellulose (NFC) which are used in biomedical applications. Various mechanical shearing methods or a combination of both chemical and mechanical methods can be used to obtain NFC. For example, Surip et al. [33] had produced NFC of 40–200 nm in diameter from kenaf fiber which was treated with alkali followed by acidic treatment (HCl) before it was mechanically processed by using pulverisette and cryo-crushing. In general NFC is a material composed by nanosized cellulose fibrils with a high aspect ratio (length to width ratio). Fibril widths can be as small as 3–10 nm but typically the size ranges between 20–40 nm with a wide range of lengths up to several micrometers [34]. Finally, the most interesting NFC material is the one produced by several bacterial strains which is referred to as BC (see above). BC is a pure cellulose and therefore no chemical treatments are required to remove lignin and hemicelluloses, as is the case for PC. For these reasons, BC is often preferred to cellulose obtained from other sources. However, reports exist suggesting that plant derived cellulose is better since BC may have inconsistent structural morphology and the encapsulation of substances may be difficult [35].

### 4.2. Cellulose Nanocrystal (CNC)

When subjected to strong acid hydrolysis, macroscopic or microscopic forms of cellulose undergo transverse cleavage along the amorphous regions resulting in the production of a rod-like material referred to as cellulose nanocrystal (CNC) [36]. The typical diameter of CNC is around 2–20 nm with a wide range of length distribution from 100 to 600 nm [37]. CNC has attracted a spectacular interest in the field of nanocomposites due to unique properties such as high surface area (and a surface area of several hundred meter per each gram) [38], low density (estimated to be 1.61 g/cm^3^) [4], readily available, renewable, and biodegradable. The impressive mechanical strength with a Young’s modulus around 150 GPa makes CNC a desirable and highly potential candidate as green reinforcing material for composite [39,40,41].

Sulfuric and hydrochloric acids have been extensively used for CNC preparation. The use of the two different acids produces CNC with different properties. If CNC is prepared by hydrolysis in hydrochloric acid, its ability to disperse in aqueous medium is limited and it tends to flocculate [42]. In contrast, the use of sulfuric acid promotes the dispersion of CNC in water. This may be due to the reaction on the surface hydroxyl groups of cellulose that yield charged surface of sulphate esters. However, the introduction of charged sulfate groups compromises the thermo-stability of the nanocrystals) [43]. Another difference observed between the two acids is the rheological behavior. Suspensions obtained from sulfuric acid hydrolysis have shown no time-dependent viscosity. In contrast, the hydrochloric acid-treated suspension showed a thixotropic (time-dependent shear thinning) property behavior at concentrations above 0.5% (w/v) and antithixotropic behavior at concentrations below 0.3% [42].

The acid concentration, hydrolysis time and temperature variation produce CNC with different morphology and characteristics as summarized in Table 1. CNC has the potential to be used as reinforcing materials in structural composites industry to fabricate building boards. However several attempts had been made to introduce CNC in the biomedical field (see Section 6) for example to improve the mechanical properties of hydrogel used for drug delivery [44,45].

### 4.3. Microcrystalline Cellulose (MCC)

The strong hydrogen bonding between individual CNC promotes re-aggregation during spray-drying procedures [58]. This event leads to the formation of another cellulose structure called microcrystalline cellulose (MCC). The length dimension of MCC is generally greater than 1 µm. MCC is stable, chemically inactive, and physiologically inert with attractive binding properties [11]. Commercially available MCC is used: (1) as a rheology control agent; (2) as a binder in the pharmaceutical industry [59]; (3) as a tablet binder and in food applications and (4) as a texturizing agent and fat replacer [11].

### 4.4. Chemical Modification of Nano Cellulose

Nano cellulose chemical modifications, which include esterification, cationization, carboxylation, silylation and polymer grafting [8], can produce derivatives with wider applications. During modification, certain functional groups are introduced with the aim to: (1) increase the amount of stable positive or negative charges for better dispersion; (2) improve compatibility, especially when used in conjunction with nonpolar or hydrophobic matrices in nanocomposites [11]. Among most common chemical modifications there are acetylation and carboxy-methylation as below detailed. Cellulose acetate (CA) is produced by using acetic anhydride in acetic acid [60,61,62]. Carboxymethyl cellulose (CMC) is produced when the hydroxyl groups is substituted with the carboxymethyl group [63]. CMC is the most important water-soluble cellulose derivative, which finds many applications in the food industry, in cosmetics, in pharmaceuticals and many more [64].

Whereas the above reported chemical modifications can improve nanocellulose properties in relation to the specific application, there are some points to be considered with regard to their biomedical use. First, derivatization can make the produced material more expensive. Second, chemical modifications can make cellulose “less natural” and therefore less likely to be accepted by regulatory agencies. With regard to this last aspect, it has to be pointed out that limited data regarding the toxic potential of such modifications are available. A recent work [65] elegantly explored in the embryonic zebrafish animal model, the potential toxicity of cellulose modifications. Compared to previous tests limited to the analysis of the effects at the cytological level, this work explores the effects on a more complex and informative model of developing vertebrate. Among others, the authors evaluated the effects of the addition of carboxyl, ethoxyethanol, ethylenediamine, hexamethylenediamine and sulfur groups to cellulose. The results indicated that the chemical modification considered induced relatively low incidences of mortality or any other developmental impairment when the embryos were exposed to doses below the relatively high concentration of 1000 mg/L for 5 days. These data seem to support no significant toxicity in the animal model considered, at least with regard to an acute exposure. Obviously, the effects of longer time exposure need to be further investigated. Despite this, the substantial lack of major adverse effects in an animal model and the known biocompatibility of nanocellulose [7] makes this material with and without chemical modification attractive form many biomedical applications.

## 5. Biomedical Applications

### 5.1. Wound Healing

**Figure 6:**
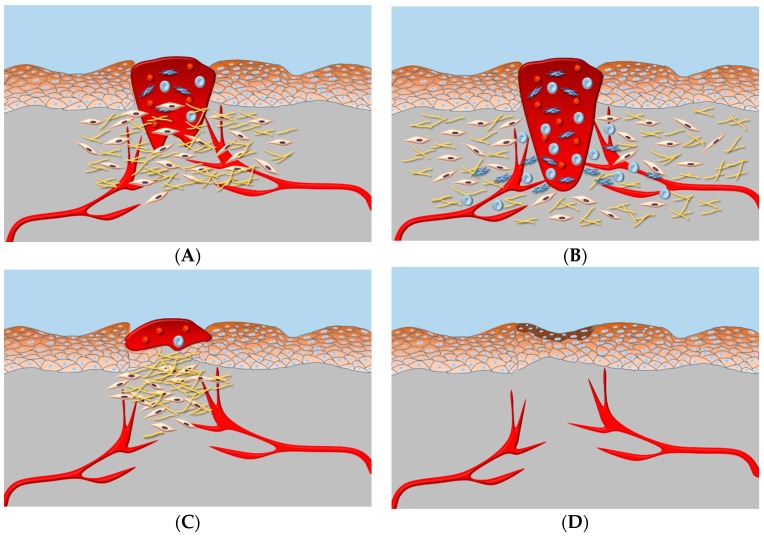
(**A**) The wound causes the formation of the clot (dark red area) and the recruitment of macrophages (blue) and neutrophils (round blue); (**B**) Fibroblasts (white) are also recruited to the wound site to fill it via collagen production (light brown); (**C**) Collagen and fibroblats rebuilt the tissue inducing endothelial cells proliferation and vessel generation; (**D**) Skin stem cells generate mature skin cells obtain the wound closing.

In the process of wound healing, a number of pathophysiological events such as tissue regeneration, repair and reconstruction take place [66]. Another relevant biological event is represented by angiogenesis, a process involving the growth of certain cell type named endothelial cells [67]. Angiogenesis is a pivotal determinant in wound repair as newly generated blood vessels allow the transport of oxygen and nutrients to cells at the site of wound [68] (Figure 6). Among wounds, those caused by thermal sources represent a critical problem for clinicians especially due to the high risk of bacterial infections. Infections can delay wound healing and can be responsible for up to 75% morbidity in burn patients [69]. Additionally, extended skin burn wounds can lead to excessive fluid loss and possibly to patient death. Thus, in burn patients, proper wound healing is crucial for survival. Critical is also the healing of wound in patients undergoing oncological treatments. In these subjects, the oncological treatments can impair tissue vitality rendering proper wound healing problematic. Finally, wound healing can be also challenging in patients with comorbidities, which can negatively affect the healing process.

Despite current advances in wound treatment, optimal approaches to promote would healing and contain bacterial infection are lacking. In this regard, the use of cellulose-based material is emerging as an attractive option.

#### Nano-Sized Cellulose-Based Material in Wound Healing

In general, the cellulose containing materials here described (Table 2) are used with the intent not to persist in the biological environment for very long time, rather to be subsequently degraded after having completed their functions.

**Figure 7:**
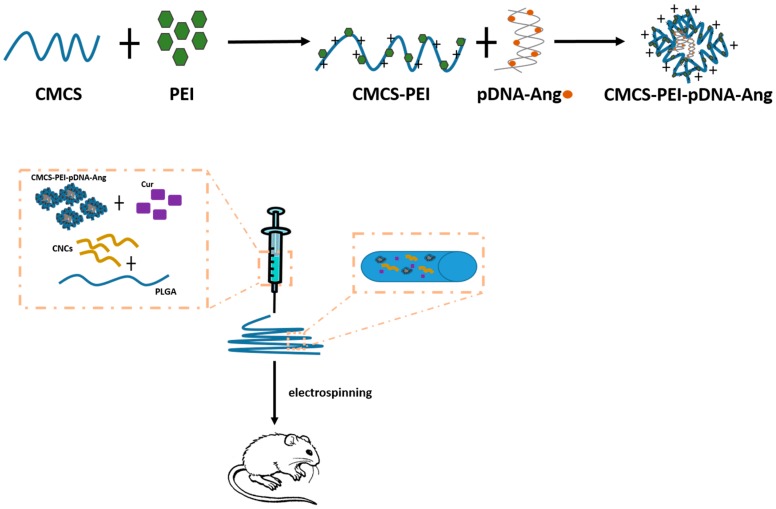
Schematic illustration of reparation of PEI-CMCS/pDNA complex nanoparticles, and assembly with Cur, CNC and PLGA; electrospinning was used to deliver the composite nanofibers to rat wounded skin. Redrawn for ref [70]. ANG = Angiogenin; CMCS = carboxymethyl chitosan; Cur = curcumin; pDNA = plasmid DNA; PEI = polyethyleneimine; PLGA = poly(d,l-lactic-co-glycolic acid).

CNC was used to generate a delivery composite containing poly(lactic-co-glycolic acid) (PLGA). This, in turn, was loaded with pre-assembled polyethylenimine (PEI) and carboxymethyl chitosan (CMCS) (PLGA/CNC/CMCS) containing curcumin (Cur) and the DNA encoding for angiogenin (ANG) [70] (see also Figure 7). CNC, obtained by acid hydrolysis from MCC, was used to confer to the delivery complex improved mechanical properties. PLGA is a copolymer often used in therapeutic devices due to its biodegradability and biocompatibility [71]. PEI, a polymer with repeating unit composed of the amine group and two carbon aliphatic CH_2_-CH_2_ spacer, is often used as bio-delivery agent [71]. CMCS is a derivative of chitosan, a high molecular weight polysaccharide of natural origin often emplyed as delivery material [72]. Cur has antioxidant, antitumor, anti-inflammatory and anti-bacterial properties, which can be used to improve wound healing. ANG is an angiogenic factor that can stimulate blood vessel formation thus promoting wound repair. With regard to the delivery properties of the PLGA/CNC/CMCS/Cur/pDNA-ANG compound in vitro, Cur was released with an initial burst (24 h) followed by a slower rate that lasted up to 144 h. DNA coding for ANG was released at a rate of nearly 50% over the first 3 days and more than 90% was released over the following 21 days. Together these data support the prolonged and controlled release of the two therapeutic compounds considered, i.e., Cur and ANG. In the endothelial human cells HUVEC, PLGA/CNC/CMCS/Cur/pDNA-ANG nanoparticles induced the increase of ANG expression of about 3 times compared to empty nanoparticles. This was due to the delivery of the DNA encoding for ANG, which promoted ANG expression. Notably no significant cell toxicity was induced by the nanoparticles in vitro. In a rat model of skin full-thickness burn, compared to control, PLGA/CNC/CMCS/Cur/pDNA-ANG treated animals always had a significantly higher expression of ANG. The increased ANG expression was paralleled by an improved density of mature vessels compared to empty nanoparticles. Even more importantly, PLGA/CNC/Cur/pDNA-ANG treated animals had significantly improved wound healing 14, 21, and 28 days post nanoparticles administration compared to animals treated by empty nanoparticles.

In a variation of the above experiment, Cur was loaded into gelatin microspheres (GMs) and then mixed with a porous collagen (Coll) containing chemically synthesized CNC to form the composite scaffold (Cur/GMs/Col-CNCs scaffold) [73]. CNC was used to improve the mechanical properties of the scaffold and because of its lack of cell toxicity. In vitro, Cur release from Cur/GMs/Col-CNCs scaffold reached 100% after 240; much shorter was in contrast the release from Cur/Col-CNCs (25 h) and Cur/GMs (130 h). This indicates the prolonged and controlled delivery mediated by the Cur/GMs/Col-CNCs scaffold. Moreover, the in vitro antimicrobial activity (against *Escherichia coli*, *Staphylococcus aureus* and *Pseudomonas aeruginosa*) was remarkable for the Cur/GMs/Col-CNCs scaffold. The effectiveness of Cur/GMs/Col-CNCs scaffold was then tested in a wound-infection model based on full-thickness burn in rats, in which the dorsal skin was artificially burned and infected with *Pseudomonas aeruginosa.* The Cur/GMs/Coll-CNCs group exhibited higher epithelializing rates than the sterile gauze group, most likely because of the extended release of Cur. Moreover, the Cur/GMs/ Coll-CNCs group had the smallest skin defect and the newly generated skin was similar to the surrounding skin, accompanied with the growth of hair. This is probably due to the excellent oxygen permeability, controlled evaporative water loss, promotion of fluid drainage and maintenance of a humid healing environment compared to the sterile gauze group.

NFC of bacterial origin was used to prepare a sustained delivery matrix for the antiseptic drug octenidine [74]. The choice of NFC was based on its high mechanical and thermal stability as well as softness. Moreover, it was chosen because of its extended surface area that can hold a large amount of drug molecules. Octenidine is used for the local treatment of wounds to down regulate the development of bacterial infection. A prolonged delivery is important to control bacterial infection for the time required to the wound to heal. To obtain a prolonged delivery, NFC was combined with Poloxamers, amphiphilic surfactants composed of polyethylene oxide (PEO)/polypropylene oxide (PPO) arranged in a triblock structure as PEOx-PPOy-PEOx [75]. Polaxamers form micelles with a core of hydrophobic PPO blocks and a shell of strongly hydrophilic PEO in an aqueous environment and thus can be loaded by the hydrophilic drug octenidine. The presence of Poloxamers and NFC allowed delayed and prolonged delivery of octenidine up to one week in vitro. Moreover, the anti-bacterial activity of octenidine was maintained against the bacterial strains *Staphylococcus aureus* and *Pseudomonas aeruginosa*. Importantly, no significant in vitro cell toxicity was observed for the CNF-Polaxamers delivery matrix.

NFC of bacterial origin was also used to deliver another antimicrobial compound, i.e., zinc oxide (ZnO) [76]. Bacterial NFC was employed due to the high biocompatibility, hydrophilicity and nontoxicity, all features that in the opinion of the authors make this material ideal for wound care. ZnO is an inorganic compound with anti-bacterial properties. Whereas NFC alone did not display significant antimicrobial activity, the addition of ZnO conferred the ability to effectively down regulate the growth of different bacterial strains including *Escherichia coli*, *Staphylococcus aureus* and *Pseudomonas aeruginosa*. In vivo, in a skin burn mice model, NFC -ZnO showed enhanced wound healing and tissue regeneration activity as compared to NFC alone. Interestingly, histologic analysis revealed that in NFC-ZnO treated tissues the whole surface of wound bed was covered with regenerated epithelium. Moreover, new hair follicles were visible near to the wound area. As hair follicles are a reservoir of epithelial stem cells, their presence supports the ongoing regenerative process. Finally, new blood vessels were observed deeper in the wound tissue, further confirming the occurrence of the reparative process.

**Figure 8:**
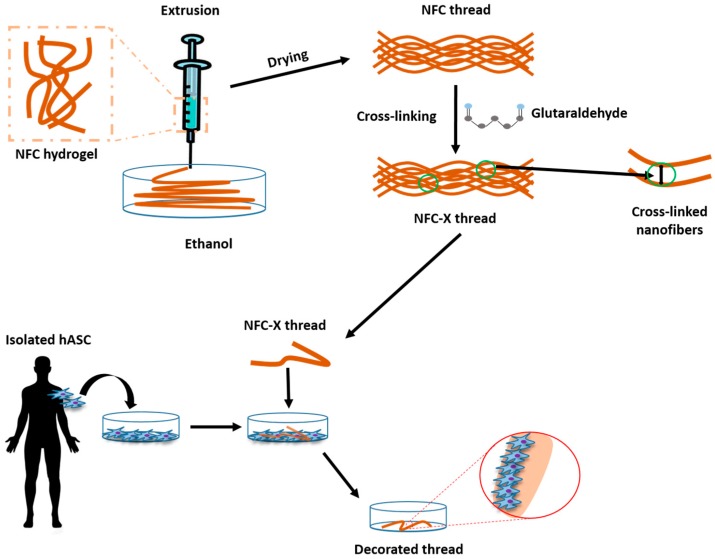
Schematic preparation of nanofibrillar cellulose with glutaraldehyde cross-links (NFC-X) threads and their cross-linking with glutaraldehyde for NFC-X threads. Following preparation, NFC-X threads were loaded with human adipose mesenchymal stem cells (hASC); cells could properly, adhere, migrate and proliferate on the NFC-X. Redrawn from ref [79].

In addition to minimize the risk of infection, wound healing can be improved via an accelerated reconstitution of the damaged tissue. This is particularly true for the surgery wounds. To promote tissue regeneration and thus wound healing, the use of human adipose mesenchymal stem cells (hASC) is considered an effective strategy [77]. However, the efficacy of any stem cell therapy depends on the ability to properly deliver and retain cells at the target area. The simple local injection of stem cells commonly results in low cell retention as most of the cells circulate via bloodstream [78]. In addition, the cell delivery/carrier material should not interfere with stem cell biology. In a recent work [79], plant NFC was used to prepare threads containing hASC to suture wounds. NFC was used because of its biocompatibility and mechanical properties. To further improve the mechanical strength, NFC threads have been reinforced by glutaraldehyde cross-links, which created bridges within different threads (NFC-X) (see also Figure 8). Notably, improved mechanical strength was maintained under the wet cell culture or surgery conditions, a challenging feature for cellulosic materials. Notably, the generated treads resulted to be mechanically robust enough to pass through different tissues (muscle, fat and skin). Moreover, hASC could properly adhere, migrate and proliferate on the NFC-X. Importantly, NFC-X threads maintained the hASC undifferentiated profile and functionality in an ex vivo suturing assay. This opens the possibility that in vivo hASC may have the ability to engraft the sutured tissue accelerating wound healing.

### 5.2. Bone Regeneration

The increase in the life expectancy together with the high incidence of diseases that cause bone loss, such as infections, tumors and trauma, makes the optimization of bone grafts technology very relevant. This necessity is particularly true in elderly people where the bone turnover is significantly decreased compared to young people.

**Figure 9:**
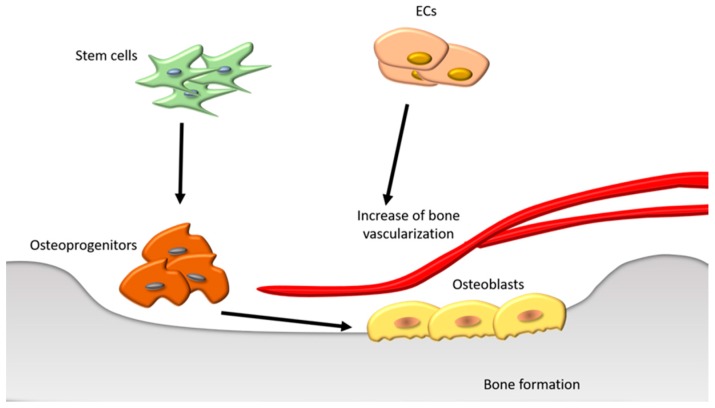
Bone formation: growth factors induce stem cells to differentiate in osteo-progenitors. These cells, in turn, differentiate into osteoblasts that attach to bone. In addition, endothelial cells (ECs) play an important role in the bone formation promoting its vascularization.

Ideally, the grafted material should: (1) promote the differentiation and growth of the main cellular component in the bone, i.e., osteoblasts; (2) guiding bone growth to the desired areas; (3) favor the integration of the new growing bone into the surrounding bone; (4) favor the deposition of calcium phosphate crystals, pivotal components of the bone; (5) favor vessel formation to nourish novel osteogenic cells (Figure 9).

As hydroxyapatite (Ha) is the main calcium phosphate phase constituent of natural bones, in principle it represents the ideal grafting material. However, synthetic Ha presents some drawbacks associated with its mechanical and chemical stability [80]. Among other materials, nanocellulose is emerging as an attracting solution for the development of novel and effective bone grafting techniques.

#### Nano-Sized Cellulose-Based Material in Bone Regeneration

As pointed out in the previous section, also in the bone regeneration field, the nano-sized cellulose used is intended to be degraded over time eventually disappearing (Table 3). Moreover, mostly chemically modified nano-sized cellulose materials have been employed in most recent works as below detailed.

**Figure 10:**
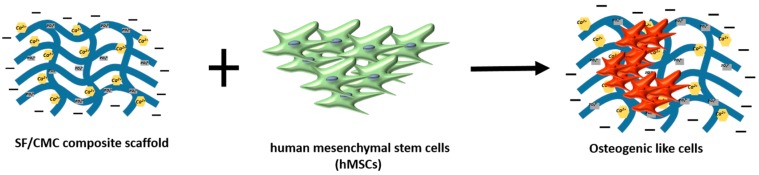
The SF/CMC composite scaffold was able to fix Ca^2+^ via chelation and to favor the homogeneous deposition and nucleation of the calcium/phosphate (Ca/PO_4_) crystals on the grafting matrix. Moreover, when loaded by hMSCs, the scaffold allowed hMCs differentiation towards osteoblastic cells. Redrawn from ref [81].

CMC [81] has been used in combination with silk fibroin (SF) to generate a bone-grafting scaffold. SF is a protein contained in the silk produced by the silkworm *Bombyx mori*. Fibroin is a biopolymer with tunable mechanical properties, a fact that makes it an important scaffold material for hard tissue engineering applications. The bone grafting material produced [81] combines the properties of silk fibroin and CMC. In particular, while SF has high mechanical strength and bio-compatibility, CMC, due to the presence of carboxymethyl groups, has hydrophilicity and the ability to fix Ca^++^ via chelation. The Ca^++^ fixing ability favors the homogeneous deposition and nucleation of the calcium/phosphate (Ca/P) crystals on the grafting matrix (see also Figure 10). The SF/CMC scaffold was loaded with human mesenchymal stem cells (hMSCs) to verify the ability to allow differentiation towards the osteogenic lineage. The combined SF/CMC material showed excellent physicochemical features together with the ability to allow the uniform deposition and nucleation of Ca/P over the surface of the scaffold. Moreover, the SF/CMC scaffold displayed improved osteoblastic differentiation of hMSCs compared to the pure silk fibroin and gelatin matrix used as controls. Enhanced hMSCs differentiation was also proven demonstrating the improved expression of the osteoblastic differentiation markers alkaline phosphatase and RUNX2, compared to gelatin. Together the above data indicate the great potential of the SF/CMC scaffold for bone regeneration.

CMC has been also used to prepare microparticles for bone regeneration purposes [82]. Microparticles can be used both as a temporary support for cells attachment/proliferation and as delivery vehicles of bioactive agents. CMC has been combined via ionic crosslinking with the aqueous ion complex of zirconium (Zr) and further complexed with chitosan (CS). Due to the biocompatibility and the negative charge present on the carboxyl group, CMC has been considered by the authors particularly suited to bind the positively charged Zr atoms. Zr has been used due to the good mechanical properties and corrosion resistivity. Moreover, Zr in culture improves the differentiation and proliferation of human osteoblasts [83]. CS is a high molecular weight polysaccharide of natural origin; it is often used as delivery material due to the possibility of the easy chemical modification of its structure thus obtaining molecules with the desired chemical-physical features [72]. Zr microparticles were prepared with two different concentrations of Zr (5% w/v-P1 and 10% w/v-P2). Microparticles had a size that ranged from 700 to 750 μm and a spherical shape. When incubated in physiological solution for 30 days, P1 lost its structural integrity while P2 maintained it. Both P1 and P2 microparticles induced negligible cytotoxicity when put in contact with the murine pre-osteoblasts (OB-6) cell line in culture. Moreover, OB-6 could proliferate on the surface of both P1 and P2. However, a significant difference was observed on the area covered by cells: about 20% and 6% of P2 and P1 surface area, respectively, were covered. This suggests that higher amount of Zr on P2, promoted cell proliferation more vigorously. The authors also investigated the delivery ability of the microparticles when loaded with cefazolin, a model drug used in the treatment of bone diseases associated with bacterial invasion. P1 and P2 showed similar release kinetics with an initial burst release followed by slow release. By day 27, P2 and P1 released 35% and 50% of encapsulated drug, respectively. It is possible that the above reported loss of the structural integrity of P1 in physiological solution, might have led to the higher release compared to P2. Together these data suggest P2 as a better candidate for bone regeneration purposes both in terms of induction of osteogenic cells growth and of the possibility to have long lasting drug delivery. Experiments in animal models are now necessary to fully elucidate the potential of the P2 microparticles.

Cellulose obtained from cotton has been combined with nano-(Ha) to prepare scaffolds for bone tissue engineering [84]. The authors preferred the use of plant cellulose as they assert BC often has inconsistent structural morphology such as fiber diameter and the inability to encapsulate substances. Additionally, no details about cellulose structure (CNC, NFC) have been provided. The morphological evaluation of the fibers constituting the scaffold revealed that the average fiber diameter was dependent on the presence of nano-Ha. In particular, the diameter increased with the increase of nano-Ha amount in the scaffold. Notably, the diameter range fall within the diameter distribution of natural extra-cellular matrix (ECM) fibers (50–500 nm), thus proving the similarity with the physiological ECM. With regard to the mechanical properties of the scaffold, they were also influenced by the amount of nano-Ha. In particular, at low nano-Ha concentration (below 5%) the strength was maximal and progressively declined increasing nano-Ha concentration (10%). This behavior was attributed to the fact that at high nano-Ha concentration (10%), the formation of a large number of nano-Ha agglomerates occurred. These agglomerates were considered responsible for the formation of “defects” in the fiber, which impaired fiber strength. Nano-Ha also improved scaffold thermal stability most likely because nano-Ha served as heat isolator. Finally, scaffold toxicity was tested in primary human dental follicle cells (HDFCs). Whereas not specified in the work, these cells were probably used are they are considered progenitor cells able to differentiate towards the osteogenic lineage [85]. The ability of HDFCs to adhere and proliferate on the scaffold was directly proportional to the nano-Ha percent. In particular, using 3% nano-Ha, cells were viable but did not well spread; cell proliferation obviously increased using nano-Ha at 5% and it further progressed with the increase of nano-Ha content, reaching the optimum at 10% when cells reached confluence on the scaffold. This indicates that nano-Ha positively affects cell behavior on the scaffold. The fact that at 10% nano-Ha cell compatibility is maximal but the mechanical properties are not, suggests that the amount of 5% nano-Ha may not be exceeded in the preparation of scaffold to be tested in more complex animal model of bone regeneration.

CA has been combined with pullulan (P) to prepare a 3D bone grafting scaffold [86]. P is a natural linear polymer composed of glucose residues polymerized into repeating malto-triose units [72], with the ability to promote osteogenic differentiation in vitro [87]. Moreover, P provides 3-dimensionality to the scaffold, a required property for bone scaffolding. CA has been chosen because of the excellent mechanical properties that can confer to the scaffold the necessary strength, one of the main properties of natural bone tissue. Moreover, the physical properties of CA well mimic those of bone microenvironment [88] thus supporting osteoblast growth in vitro and bone formation. Finally, following scaffold degradation, CA can be easily eliminated by the kidney. Optimal ratio (%) P/CA was found to be 50/50. At this ratio, the scaffold consisted of more homogeneous fiber size and morphology compared to different ratios; these features make the 50/50 ratio suited to host osteogenic cells. The scaffold was further reinforced by the crosslinking with trisodium trimetaphosphate. Moreover, the crosslinking made the surface of the scaffold fibers more stable and thus more suited for mineral deposition. With regard to the porosity, the scaffold had pores within 100 and 400 μm, thus in line with the porosity necessary for bone formation. The presence of the large pores allows the formation of new bone and capillaries necessary for the successful diffusion of nutrients and oxygen within scaffold. Experiments conducted in vitro in the human Osteogenic Sarcoma Cell Line (Saos-2) confirmed these predictions. Saos-2 cells proliferated up to day 7 after seeding in the scaffold. Notably, proliferation was not limited to the scaffold surface, it occurred also at 210 μm from the surface, most likely because of the large porosity of the scaffold that enabled nutrient/oxygen transfer and thus cell penetration. At day 14, cells became spread and stretched from a fiber to another, exhibiting filopodia-like features among fibers thus suggesting a certain degree of osteogenic differentiation. Differentiation was confirmed by evaluating the levels of Alkaline Phosphatase (ALP), a known marker of osteogenic differentiation. Notably, ALP level was three times higher than that measured for the same cell line seeded onto a common polystyrene surface. The author speculated that such increase might be due to the 3-dimensional fibrous network that better mimics the structure of the ECM. Together the above data suggest that the crosslinked scaffold has the potential to effectively allowing bone regeneration, a feature that however needs to be confirmed in animal models.

(Hydroxypropyl)methyl cellulose (HPMC) in the form of gel has been used to deliver calcium (Ca) in the form of Ca phosphate (CaP) glass-ceramic particles into the bone [89]. HPMC has been undertaken as it exhibits suitable biological characteristics and it has already been successfully used for the delivery of CaP ceramics [90]. Calcium release has the ability to promote vascularization [91] when delivered in a controlled fashion. Indeed, Ca can induce the migration, maturation and organization of endothelial progenitors [92]. Improved vascularization is of particular relevance for bone regeneration. This aspect is of great relevance in the case of large bone defects, where oxygen may not reach osteogenic cells far from the supplying blood vessels; indeed, passive oxygen diffusion is limited to about 150 and 200 nm [93]. The injectable hydrogel HPMC-CaP was first tested in cultured human progenitor-derived endothelial cells (PDECs). In this model, HPMC-CaP did not show any significant toxicity and promoted the expression of vascular endothelial growth factor (VEGF), a signal protein able to stimulate vasculogenesis and angiogenesis. In vivo, HPMC-CaP was tested in a rat bone defect model. In particular, 4 mm diameter and 6 mm depth holes were created in both left and right femoral condyles of rats. Created holes were then filled with HPMC-CaP and bone regeneration was evaluated at 3 and 6 weeks post-implantation. Compared to HPMC alone, HPMC-CaP showed increased bone regeneration as evaluated by histology and micro CT. Notably, a significant increase in vessel density was observed in HPMC-CaP compared to HPMC treated animals. Together, the presented data indicate that HPMC-CaP can promote both osteogenesis and vasculogenesis thus representing an attracting material for future studies in the field of bone regeneration.

### 5.3. Cartilage Regeneration

Once degenerated, wounded or affected by genetic defects, cartilage has limited ability to heal and/or regenerate, because it is an avascular tissue. Unfortunately, current reconstructive options for its repair or replacement are limited. A recent novel option for cartilage replacement is represented by the bioprinting technology. This technique can generate 3D tissue via the layer-by-layer deposition of a scaffold structure containing the appropriate cell type. A critical aspect in the generation of printable structures is represented by use of appropriate bioink that should have appropriate viscoelastic properties required for printing fidelity and long term structural stability. As an alternative to bioink, there is the possibility to generate multiple layer scaffolds.

#### Nano-Sized Cellulose-Based Material in Cartilage Regeneration

A bioink based on NFC and alginate (A) has been used to perform the bioprinting technology [94]. Alginates, a family of unbranched polysaccharides isolated from brown seaweeds [95], are composed of 1-4-linked β-d-mannuronic acid (*M*) and alpha-l-guluronic acid (*G*) arranged in a block-wise pattern. In the presence of Ca, the different chains of alginates crosslink each other giving origin to strong and permanent structures. Thus, the generated bioink combines the shear thinning properties of NFC with the cross-linking ability of A. In particular, a bioink containing 80/20 (w/w) of NFC and A, has the appropriate features in term of rheology, compression and shape deformation. The NCF/A bionk was embedded with human bone marrow–derived stem cells (hBMSCs) and human nasal chondrocytes (hNC) [96]. hBMSCs and hNC were used together as their combination enhances chondrogenesis [97]. To evaluate neocartilage formation and the stability of the bioprinted constructs in vivo, the NCF/A loaded by hBMSCs/hNC was implanted subcutaneously on the back of 6-week-old nude female mice. Wounds were then sutured and covered with a sterile wound tape. After 60 days, the formed tissue had all the qualitative features of proper cartilage; moreover, the formation of chondrocyte cell clusters clearly indicated the ability of embedded cells to proliferate. Notably, cell clusters contained human chromosomes proving their human origin. Together, these data indicate the feasibility of cartilage synthesis in constructs with high fidelity and appropriate mechanical and biological properties. Finally, the authors affirm that occurrence of a good host tissue integration in vivo, thus suggesting the long term permanence of the bioink.

An alternative to the use of bioink for cartilage generation is the preparation of layered scaffolds. In this regard, bacterial NFC has been used to prepare a bilayer scaffold composed of a dense layer joined with a composite layer [98]. NFC was used as it is composed by highly hydrated nanofibrils ranging from 70 to 140 nm in width that resemble the structure of collagen fibrils found in extracellular matrix of several tissues. Moreover, NFC has the tensile strength required for the specific application. The dense layer, containing NFC at 17%, was generated by compression; the porous layer containing NFC and A, was obtained by a freeze-drying process. To tightly fixing the two layers, the cellulose solvent system “ionic liquid EMIMAc”, was used. The composite layer was generated with macro porous. Macro porosity is necessary to support cell ingrowth and neo-cartilage formation. Indeed, NFC tend to be a too dense network which obstacles cell penetration and homing. The macro porous layer of the scaffold was then loaded with human NC and cultured in vitro for up to 6 weeks. Following this examination time, the adhesion between the two layers remained good and the size/shape of the scaffold were maintained. Moreover, the porous layer supported NC growth also facilitating NC homogeneous distribution. Importantly, NC were able to produce and accumulate cartilage-specific ECM components in the scaffolds. This observation, together with the upregulation of the expression of chondrogenic markers, witnesses the suitability of the scaffold to promote chondrogenesis. Based on these promising in vitro results, the scaffold was implanted subcutaneously in nude mice. In addition to NC, the scaffold was loaded also with bone marrow mononuclear cells (MNC), which are known to improve cellular grafting [97]. As control, the scaffold without cells was implanted. Eight weeks post-implantation, a thin fibrous capsule surrounded the MNC/NC-scaffold and the cell-free scaffolds. This was considered a normal non-pathological foreign body reaction. The shape and size of the scaffold remained stable and no delamination between the two layers was observed. The scaffold seeded with MNC and NC had a macroscopically cartilage-like appearance. Notably, the MNC/NC-scaffold was stiffer and more stable, compared to the cell free scaffold. Together these observations indicate the development of a mechanically stable scaffold able to maintain a long-term structural integrity. In conclusion, the data described strongly support the suitability of the use of NFC to generate scaffold for cartilage (re)-generation (Table 4).

### 5.4. Dental Applications

In contrast to the wound healing and bone regeneration fields, the cellulose containing materials used in most dental applications have to persist as much as possible at the application site (Table 5).

For the strong mechanical properties, nano cellulose has been employed as filler in many dental materials and composites. Glass ionomer cement (GIC) is frequently used in restorative dentistry and its properties have been constantly improved because of its significant flexibility in clinical applications. Even though GIC cement has been indicated by international bodies as the definitive restorative material, scientific investigations are still evolving to improve its wear resistance, compressive and diametric tensile strength. New GIC formulations are being developed to make use of NFC as a reinforcing agent in dental restorative material. Recently, Silva et al. [99] modified conventional GIC by NFC from eucalyptus pulp. The mean values of the length (L) and diameter (D) of the isolated NFC were determined to be 145 ± 25 nm and 6 ± 1.5 nm, respectively, with an aspect ratio (L/D) of 24. The surface of the GIC-NFC 0.4% (G04) showed the formation of a network formed by long fibers with uniform width and random distribution within the matrix. Moreover, it was also found that the addition of NFC increased substantially all the mechanical properties of the generated material. The compressive strength and the diametric tensile strength, for example, increased from 49.15 MPa and 28.27 MPa to 103.22 and 43.16 MPa, respectively. Additionally, the elastic modulus increased from 512.61 MPa to 1337.75 MPa.

Reinforcing fibers were also introduced to the family of denture based materials namely poly(methyl methacrylate) (PMMA), in order to fulfill their requirement of higher mechanical properties against occlusal overload. Among the wide varieties of reinforcing agents used, glass fibers [100,101] and several kinds of polymeric fibers for example ultra-high molecular weight polyethylene fiber (UHMWPE) [102] were employed. Preliminary studies investigating the potential of ramie fiber as reinforcing agent for denture based resin has been carried out. Ramie, known as “china grass” and referred to as the best fibers, are extracted from the phloem tissue of the plant. Although the cellulose fibers were not in the nano-sized scale, the addition of natural fibers was found to improve the mechanical properties. In general, this modified denture composite had higher flexural modulus than neat resin but the flexural strength declined due to the weak interfacial adhesion [103]. In this case, the incorporation of NFC will probably improve the interfacial bonding between the fibers and the surrounding matrices.

Aside from prosthesis, NFC can be also incorporated in scaffold materials for various dental procedures. Regenerative dental treatment refers to the restoration of supporting tissues of the teeth such as bone, cementum and periodontal ligament to their original healthy levels [104]. Bone grafts are applied with the use of barrier membranes that offer better protection and containment of the bone substitute inside the defect. There are two types of membrane: (1) resorbable such as collagen and polylactic acid; (2) non-resorbable membrane materials such as ethyl cellulose. Barrier materials in the form of semipermeable membranes are placed between the mucoperiosteal flap and the bone and tooth surfaces during surgery [105]. These barrier membranes can make it possible to prevent the fast-growing gum tissue from getting into the regenerative site, a fact that might interfere with the process of bone grafting [106].

For the successful application of scaffolds in tissue engineering, a crucial feature is that the matrices promote cell adhesion and migration. Takata et al. [107] previously studied the migration of osteoblastic cells on various guided bone regeneration membranes. Among the membranes tested there are the bovine type I atelocollagen (Tissue GuideA; TG, Koken, Tokyo, Japan), polylactic acid (Epi-GuideA; EG, THM Biomedical Inc., Duluth, MN, USA) and co-polymer of cellulose acetate and nitrocellulose (Millipore filter^®^; MP, Millipore Co., Billerica, MA, USA). Millipore filter (MP) containing cellulose showed uniform and enhanced cell migration compared to polylactic acid membrane. Moreover, MP showed the greatest amount of cells attached as compared to other membranes. Based on these preliminary data it appears that MP may be particularly useful to guide bone regeneration [107].

BC was tested in dental tissue regeneration [108] using the “otolith/collagen/BC nanocomposites” as a scaffold for bone regeneration. BC produced by the Gram-negative acetic acid bacteria *Gluconacetobacter xylinus*, consists of an ultra-fine gel network of cellulose nanofibres (3–8 nm). The “otolith/collagen/bacterial cellulose nanocomposites” was fabricated by immersion of dried BC into nanotolith gels and posterior soft drying at 50 °C for 12 h. Cell adhesion was verified by 4,6-diamidino-2-phenylindole (DAPI) staining. The results showed that the fermentation process and nanotoliths agglomeration decreased initial human dental pulp stem cell adhesion. However, it increased cell viability over time and demonstrated the potential of being an effective scaffold for bone and tissue regeneration [108].

### 5.5. Other Applications

In general, the nano cellulose containing materials employed in the applications below described (Table 6) are used with the intent not to persist in the biological environment for very long time, rather to be subsequently degraded after having completed their function.

#### 5.5.1. Cancer Diseases

In a recent work [109], NFC aerogel has been loaded with the hydrophilic drug bendamustine, a nitrogen mustard compound that functions as an alkylating antineoplastic agent. The drug is used in the therapy of chronic lymphocytic leukemia, multiple myeloma, and non-Hodgkin’s lymphoma. NFC was used because of the good flexibility, elasticity, low toxicity, the ability to swell in water and to host hydrophilic compounds. For NFC hydrogel, swelling was pH dependent being maximal at pH = 7.4 and minimum at 1.2 in vitro. Notably the increased swelling observed at pH 7.4 was associated with increased drug release. The authors also observed that the drug loading capacity directly correlates with the fiber concentration in the hydrogel. Moreover, in rats, following oral administration, it was observed that the plasma concentration greatly differed over time between the marketed formulation of bendamustine and the one newly developed. In the first case, the drug reached a concentration peak after less than an hour and almost completely disappeared within 5 h from administration. In contrast, with the NFC formulation, the peak was reached about 5 h after administration and detectable levels of drug were present up to 24 h after administration. This kinetic profile clearly shows the great potential of NFC hydrogel in ameliorating drug release kinetic. These data open the possibility to prepare a long lasting/controlled delivery approach for the antineoplastic agent bendamustine and possible other similar drugs.

There is a great interest in the development of colon-specific drug delivery systems for the treatment of many colon-related diseases such as colon cancer and inflammatory diseases. Usually, films based on natural polymers are employed. However often these films suffer from poor mechanical barrier and thermal properties. To overcome these limitations, recently it has been proposed the use of plant NFC. In particular, NFC has been undertaken to reinforce films made by resistant starch (RS) and Pectin (P), a natural hetero-polysaccharide found in different plant species [110]. As delivered drug, it has been considered Methotrexate (MTX), a poorly water-soluble drug, with important therapeutic effects on bowel inflammatory diseases and colorectal cancer. The addition of NFC to RS/P significantly improved film strength; this fact can be related to the intrinsic rigidity of NFC. Moreover, in an ex vivo bio-adhesion test employing porcine colonic mucosa sections, NFC improved film muco-adhesion. In addition, NFC improved the barrier properties towards water penetration compared to control films; in particular, water permeability was about four times lower. The reduction in water permeability is a desired feature as high permeability can negatively interfere with the film permanence on the colon mucosa and alter drug delivery kinetic. Finally, in vitro, NFC was able to increase MTX delivery from the film. This is a desirable feature as it can contribute to overcome the known oral bioavailability problems of a poorly soluble drug such as MTX.

Metastasis prevention and inhibition represents one of the major goal for any anticancer therapy. The implantation in the peri-tumor region of nanofibers mimicking the structure of the ECM proteins may decrease the risk of tumor cell evasion from their original site. This is probably because nanofiber and ECM have similar architecture and physical barrier properties [111]. Moreover, nanofibers can act as cell-adhesive ECM. Based on these considerations, NFCs were prepared and tested for their anti-metastatic potential [112]. Due to the ability to form matrices, NFCs were also loaded with the anti-cancer and anti-metastatic drug metformin (Met). In in vitro models of cell migration and adhesion, the Met-NFC gel was able to effectively reducing the migration, invasion and promoting adhesion of the melanoma cells B16F10. Notably at the concentration used (17.6 mg/mL), no toxic effects of Met-NFC were observed for the non-cancerous mouse fibroblasts cells L929. Further tests in more complex systems will clarify the potential therapeutic value of the Met-NFC gel.

The above examples have considered the use of native not chemically modified nano cellulose materials. However, chemically modified nano cellulose materials have been also employed in the approach to cancer diseases. As an example, we mention in this section HPMC. Radiotherapy is commonly used for the management of malignant pelvic diseases. However, radiotherapy has high toxicity on surrounding healthy tissues such as the small intestine, colon and rectum. The intravenous injection of hASC has been observed to reduce severe colorectal lesions caused by ionizing irradiation. However, a large number of hASC are necessary to obtain beneficial effects. Indeed, among the huge amount of injected cells, only a minor fraction reaches and engrafts the irradiated intestine [113]. To optimize hASC local delivery, hydrogels have been considered particularly suited. A hydrogel has been prepared by combining silane, an inorganic compound, with HPMC (Si-HPMC) [114]. The presence of silane was required to confer to the hydrogel the rheological properties suitable to allow administration via colonoscope. HPMC has been undertaken because of its suitable biological characteristics and the ability to host human cells. The Si-HPMC hydrogel is biocompatible, maintains hASC phenotype, viability and protects hASC from the deleterious irradiated environment. Moreover, in a rat model of radiation-induced severe colonic damage, hASC/Si-HPMC delivered via a colonoscope catheter, improved colonic biological conditions compared to hASC injected intravenously or locally.

#### 5.5.2. Other Diseases

**Figure 11:**
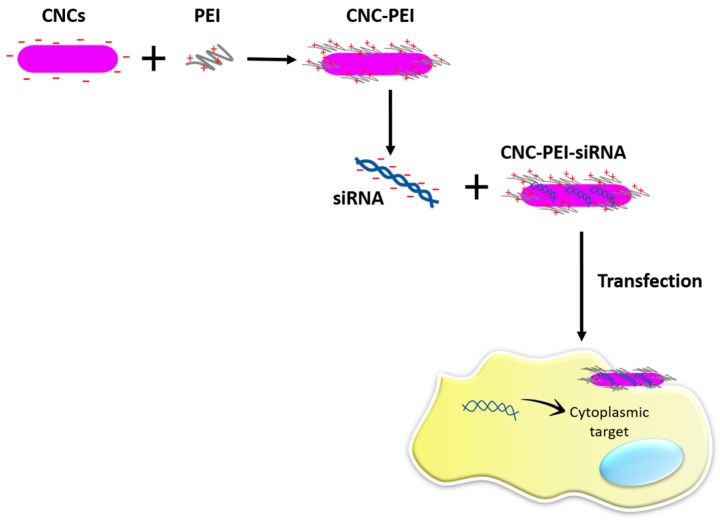
General procedure for the synthesis of CNCs-PEI-siRNA complexes and siRNA targeting in cells. Redrawn from ref [126].

Small interfering RNAs (siRNAs), belongs to the class of nucleic acid based drugs (NABD), biological molecules with high therapeutic potential [115,116,117,118,119,120,121,122]. siRNAs are short, double stranded RNA molecules (~22 nucleotides) of natural origin that can be also artificially generated. siRNAs have the ability to specifically target RNA molecules thus inducing their degradation [123]. When directed against disease-causing RNAs, siRNAs display potent therapeutic effects. Thus, siRNAs represent novel biological drugs with a remarkable therapeutic potential. However, their chemical nature does not allow the delivery to the diseased cells as naked molecules. Indeed, in the naked form, these molecules are rapidly degraded in the extracellular and intracellular environment [124]. Thus, delivery systems able to protect them from degradation are necessary to allow their therapeutic action [71,125]. A novel system based on the use of CNC has been recently developed [126] for siRNA delivery. Plant derived CNC has been chosen for being biodegradable, non-toxic and for being able to target tumors via the EPR effect (Enhanced Permeability and Retention) [127]. The presence of negative surface charges on CNC limits the interaction with the negatively charged siRNAs. To overcome this limitation, CNC has been covalently attached to PEI. The great number of positive charges of PEI, which largely surpassed the negative charges carried by CNC, allowed the electrostatic interactions with the negatively charged siRNAs (see Figure 11). Moreover, PEI enhances the uptake of associated particles into cells by endocytosis. The generated CNC-PEI nanoparticles provided efficient protection of siRNA vs. degradation. Additionally, they allowed efficient uptake in C2C12 murine myoblast. Moreover, the CNC-PEI nanoparticles carrying a siRNA that targets a cell cycle promoting gene, were able to effectively down regulating the growth of C2C12. Future studies in more complex animal models will contribute to fully determining the delivery effectiveness of this CNC-based delivery system for siRNAs. Obviously, the proposed delivery system loaded with anti-proliferative siRNAs has the potential to be used in the many forms of human diseases that bases their pathogenicity on the exaggerated cell proliferation.

In addition to the systemic delivery of siRNAs above presented, also local delivery is of therapeutic interest. For example, recently, it has been developed a siRNA topical vaginal sustained delivery system [128]. This local delivery presents a significant challenge due to the short residence time of formulations. Thus, a system capable to adhere to the vaginal mucosa is necessary to prolong delivery and increase the effectiveness of the therapy. To this end, a model siRNA was complexed into liposomes, lipid particles able to effectively protecting siRNA from degradation and to promote cell uptake [129]. However, liposomes alone are not suitable for efficient siRNA delivery due to the poor ability to penetrate through the vaginal mucus and the short residence time. To minimize the first limitation, liposomes have been coated by polyethylene glycol (PEG). This is a neutral hydrophilic polymer able to allow efficient mucus penetration due to the ability to reduce adhesive interactions between nanoparticles and mucus components. To improve the residence time, liposome-PEG compounds were incorporated into a hydrogel constituted by hydroxyethyl cellulose (HEC), a material chosen for its muco-adhesive properties [130]. Notably, HEC concentration in the gel containing liposome-PEG-siRNA, was directly correlated with the muco-adhesive properties. In contrast, liposome-PEG-siRNA did not influence the muco-adhesive capacity. Additionally, gel hardness was directly correlated with HEC concentration but not with liposome-PEG-siRNA amounts. In an in vitro delivery test, high HEC concentration (1.67% HEC) resulted in a delayed delivery compared to low concertation (0.83% HEC). This indicates the possibility to tune delivery in relation to the desired kinetic. Whereas future in vivo studies are necessary to fully elucidating the potential of the developed delivery system, it is clear the important contribution of HEC in this kind of delivery strategy.

Acyclovir is an antiviral drug able to down regulating herpes simplex virus (HSV) replication. HSV is typically responsible for different form of human diseases such as herpes labialis and herpes zoster. To get the desired therapeutic effects, acyclovir has to be administered via oral/systemic route using multiple administrations of large doses, which are often plagued by significant side effects. Recently, to optimize acyclovir delivery it has been developed a novel hydrogel composed by CMC, β-cyclodextrin (β-C) and acrylic acid (AA) [131]. CMC has been undertaken due to its excellent biocompatibility, biodegradability, low immunogenicity and ability to swell. Β-C are natural, cyclic oligosaccharides, composed of between six and eight glucose units [71,72]. They are used as drug carriers especially due to the ability to host a large number of drug molecules. AA has remarkable bio-adhesion, pH responsive and mechanical properties. The authors observed that hydrogel swelling, one of the most relevant property for controlled drug delivery, was dependent on CMC amount. In particular, swelling was maximal using a CMC amount in the hydrogel of 12 (%w/w). Moreover, swelling was pH dependent being maximal at pH = 7.4 and minimum at 1.2 in vitro. Despite no experiments have been performed in living models, it is assumable that following oral administration, maximum swelling and thus acyclovir delivery occurs in the small intestine. Here, the more basic pH compared to the stomach, should favor acyclovir release from the hydrogel.

### 5.6. Clinical Trials

Applications of nano cellulose are not limited to preclinical testing, examples exist (Table 7) of the close use in the clinic as shown by the clinical trials below reported.

Nanoparticles of appropriate sizes and stability can be pushed into hair follicles via a gear pump mechanism [132]. Thus, such nanoparticles can potentially be used to deliver anti acne drugs directly to the acne-affected follicles. To achieve this goal, ethyl cellulose-methyl cellulose (EC-MC) has been used to fabricate nanoparticles able to deliver the anti-acne compound α-mangostin [133]. EC-MC was used because of the very low price, biocompatibility and the possibility to fabricate nanoparticles with excellent drug loading capacity [134]. When embedded into EC-CM, α-mangostin was released in synthetic sebum up to 168 h with 50% delivery being reached after about 8 h.

Importantly, EC-CM nanoparticles did not trigger any significant skin inflammatory reactions as evaluated by a two-week, twice-daily application in 20 healthy human volunteers. Successful delivery of α-mangostin was proven following drug quantification in the roots of hairs obtained from the skin of six human volunteers topically treated. Finally, following a 4 week-randomized, double-blind, placebo-controlled study in 10 acne patients, it was possible to observe significant improvement in acne in correspondence of the portion of skin treated by the α-mangostin containing EC-CM nanoparticles.

The middle-ear packing material represents one of the key elements in the success of tympanomastoid surgery. This material provides support for tympanoplasty graft. Often, resorbable gelatin is used for this purpose. However, adverse effects such as fibrosis resulting in suboptimal hearing outcomes, plagues the use of gelatin. To overcome this problem it has been proposed the use of sodium hyaluronate (HA) and CMC [135]. HA is an inert and safe glycosaminoglycan easy to use and able to remain at the desired biological site for long period. CMC has been used as viscosity thickener of the emulsion of HA-CMC. Notably, HA-CMC is bioresorbable and can significantly reduce the postoperative adhesion. HA-CMC has been added to Gelfoam, a sterile compressed prepared from purified porcine skin, Gelatin and water. One hundred forty three patients with chronic otitis media that underwent tympanoplasty, were treated with Gelfoam soaked in HA-CMC; another 144 patient were instead treated with Gelfoam alone. The result of this clinical trial indicates that HA-CMC-Gelfoam treated patients had better postoperative hearing improvement and reduced complications. The authors suggested that this was most likely due to the better anti inflammatory and anti-adhesive properties of HA-CMC-Gelfoam compared to Gelfoam alone.

CMC has found also another interesting application in the preparation of artificial tears [136]. In particular, it has been studied the effectiveness of sodium hyaluronate 0.1% and CMC 0.5% artificial tears to attenuate ocular discomfort and improve tear-film stability in eyes after cataract surgery. CMC was used because of its good bio-adhesive characteristics [138] and because of its anionic nature that is beneficial in increasing tear retention time [139]. HA, a glycosaminoglycan disaccharide biopolymer composed of repeating alternating sequences of n-acetylglucosamine and glucuronate in linear chains, has the ability to bind water molecules and prevent dehydration. Two hundreds and eighty two patients were divided into two groups: after surgery, the first received artificial tears and a topical steroid-antibiotic (study group), while the second just topical steroid-antibiotic alone (control group). The patients were then evaluated post-operatively at 1 and 5 weeks by tear breakup time (TBUT) and Corneal fluorescein staining (CFS). TBUT, a clinical test used to evaluate evaporative dry eye disease, is measured following fluorescein instillation into the patient’s tear film asking the patient not to blink while the tear film is observed under a broad beam of cobalt blue illumination. The TBUT is recorded as the number of seconds that elapse between the last blink and the appearance of the first dry spot in the tear film. CFS is performed by touching a piece of blotting paper containing fluorescein to the surface of patient eye. The patient is then asked to blink to spread the dye in the tear film thus covering the surface of the cornea. By looking at the patient eye with a blue light, it is possible to detect any problems on the surface of the cornea, which will be stained by fluorescein (green dye). At 5 weeks, the mean TBUT and CFS were statistically significantly higher and lower, respectively, in the study group compared to the control group. Importantly, the hyaluronate 0.1% and CMC 0.5% artificial tears were well tolerated by the patients. Thus HA and CMC seem to be very promising components of artificial tear as also witnessed by the fact that they are the two most commonly prescribed and used substances for dry eye disorders [139].

In another interesting clinical trial, hydroxypropylmethylcellulose (HPMC) was tested in the treatment of allergic rhinitis [137]. It has been hypothesized that inhaled allergens, as well as other substances, can disrupt the integrity of nasal mucosal lining thus favoring the contact of the body with external harmful agents. Over time, this can result in the development of allergic rhinitis characterized by persistent inflammation and mucosal congestion. This in turn brings to a variable degree of nasal air flow reduction and discomfort for the patient. To down regulate inflammation and mucosal congestion, decongestant substances such as oxymetazoline nasal spray, 0.05% are often used. Recently, HPMC has been employed in addition to oxymetazoline [137] due to its known mucoprotective effects based on its viscoelastic properties. Forty allergic rhinitis patients were divided into two groups: the first was locally treated by oxymetazoline, followed by HPMC, the second was treated similarly but instead of HPMC, lactose powder (placebo) was administered. The treatment was performed twice a day for 7 days followed by oxymetazoline rescue medication for another week. Treatment effects were measured by evaluating the peak inspiratory nasal flow (PNIF) which reflects the nasal patency. Baseline PNIF was greater in the HPMC group at day 15 reflecting a more marked reduction in nasal congestion. The authors suggest that this effect may be due to the HPMC muco-adhesion properties which can improve the mucosal barrier in allergic rhinitis thus limiting the contact with allergens.

In addition to the above clinical trials, it should be also noted that BC-based materials are already on the market for different applications approved by FDA. In this regard, BC derived materials are used in vessel implants, in the fabrication of artificial skin and in wound care systems [140].

## 6. Conclusions

Due to its biocompatibility, bio-degradability, low-cost and easy availability, nano-cellulose finds application in disparate research fields. In the biomedical field, scientists can take advantage of the strength of nano cellulose that rends it suitable in different applications such as bone regeneration. In this regard, the works mentioned in the review indicate how nano cellulose and its chemical derivatives have the potential to allow the hosting of various types of progenitor stem cells. These cells can differentiate into the nano cellulose matrix giving origin to osteogenic cells thus promoting ossification. However, the beneficial effects of nano cellulose are not limited to this aspect. Indeed, nano cellulose can improve the formation of new capillaries, essential to provide nutrients and oxygen to the newly developed bone. Moreover, nano cellulose combined with other substance, has the potential to favor the deposition of calcium phosphate crystals, pivotal components of the bone.

Not only is nano cellulose suited for the (re)generation of hard tissues such as bone, when appropriately combined with other substances like alginates, it can be used to generate soft materials such as “bionk” for cartilage reconstitution. This impressive material combines the properties of nano cellulose with those of alginates giving origin to a novel compound with the appropriate features in terms of rheology, compression and shape deformation suitable to be used as bionk. Always in the field of soft tissue regeneration, nano cellulose finds potential advantages also in the wound healing field. Here, nano cellulose in combination with different materials can promote skin regeneration in different ways. In particular, it can favor hASC adhesion/proliferation, combat bacterial growth and promote angiogenesis. Together all these properties promote proper and fast healing. This is particularly important for extended skin lesion caused by thermal sources where bacterial infections and excessive fluid loss can lead to patient death if the skin is not rapidly regenerated.

Nano cellulose materials have also the potential to be used as drug delivery compounds. A noticeable example described in this review includes the systemic (oral) delivery of the anticancer drug bendamustine. The nano cellulose-based material developed may allow a long lasting/controlled delivery that may permit to improve drug effectiveness reducing side effects. In addition to systemic delivery, also local delivery has been explored with nano cellulose-based materials. Here, for example, we mention the colon-specific delivery of the anticancer drug Methotrexate. The nano cellulose-based materials in the form of gel is able to properly adhere to the colon mucosa and to create a barrier against water penetration, thus favoring gel permanence and drug local delivery.

The potential use of nano cellulose-based materials as drug delivery devices is not limited to conventional FDA approved drugs; it extends also to novel classes of drugs such as siRNAs. Despite being potentially very effective, siRNAs suffer from the poor stability when delivered into the biological environment. Nano cellulose-based materials have the ability to provide efficient protection from degradation also improving effective cellular uptake. The only drawback of nano cellulose-based materials is that they are negatively charged and thus in the native form cannot bind the negatively charged siRNAs. To overcome this limitation, positively molecules are combined with nano cellulose-based materials thus allowing siRNA binding.

Finally, nano cellulose-based materials are not far from the practical use as shown by the clinical trials mentioned in this review in different the fields such as hearing improvement, cataract surgery and the improvement of mucosal barrier in allergic rhinitis. Moreover, nano cellulose-based materials find practical application in vessel implants, in the fabrication of artificial skin and in wound care systems.

For medical applications, it seems BC to be preferable to PC as BC does not contain lignin, hemicellulose structures and its preparation does not need any chemical synthesis and/or treatments. Moreover, purified BC has a content of endotoxin falling within the acceptable range indicated by FDA. Finally, an open aspect in the use of nano cellulose materials concerns the employment of chemical modifications in relation to safety. In this respect, even if preliminary tests in animal models seem to be comforting, it will be pivotal to know how regulatory agencies will consider these materials.

Altogether, the examples of nano cellulose applications here reported strongly support the enormous application potential of this material whose relevance includes, but is not limited to, the biomedical field.

## Figures and Tables

**Table 1:** Typical geometrical characteristics for cellulose nanocrystal (CNC) originating from different cellulose sources and obtained via different procedures.

Sources of Cellulose	Hydrolysis Procedure	Nanocellulose Dimension	References
Rice husk fibers	10.0 mol L^−1^ of sulphuric acid (pre-heated) at 50 °C for 40 min under continuous stirring. 60 wt % H_2_SO_4_ solution at 45 °C for 45 min.	TEM micrograph Diameter and aspect ratio in the range of 15–20 nm and 10–15 respectively AFM micrograpgh Average diameter 12.4 ± 4.6 nm.	Johari et al. [44] Ludueña et al. [46]
*Pandanus tectorius*	60 wt % H_2_SO_4_ solution at 45 °C for 45 min.	TEM micrograph Length from 50 to 400 nm, with an average value around 200 nm. Diameter was in the range 5–25 nm.	Sheltami et al. [47]
Coconut husk fiber	64%brt H_2_SO_4_ solution at 45 °C for 30 min under continuous stirring.	TEM micrograph Average length 172.34 ± 8.4 nm. Average diameter 13.7 ± 6.2. Aspect ratio 14.23 ± 8 nm.	Ahmad and Ahmad [48]
kenaf bast fibers	65 wt % H_2_SO_4_ 120 min	TEM micrograph Average length 124.3 ± 45.3 Average diameter 11.3 ± 2.6 (nm)	Kargarzade et al. [49]
waste sugarcane bagasse	60% (w/v) sulfuric acid (fiber to liquor ratio of 1:20) for 5 h at 50 °C under strong agitation.	TEM micrograph Length and diameter: 170 nm × 35 nm.	Mandal and Chakrabart [50]
Sisal fibers	60 wt % sulphuric acid solution at 45 °C, 30 min under continuous agitation.	AFM micrographs Diameter, average size of 30.9 ± 12.5 nm	Mora´n et al. [51]
	47 wt % sulfuric acid with fiber to solution ratio of 1:20 by refluxing for 3 h at 60 °C under strong agitation.	TEM micrograph Length of 200–350 nm. Diameter of 40–50 nm	Reddy and Rhim [52]
Oil Palm Empty Fruit Bunch Fiber	64 wt % sulfuric acid solution under strong agitation at 45 °C for 45 min	TEM micrograph Diameter range of 4–15 nm	Lani et al. [53]
banana pseudostems fibers (*Musa* sp.)	50.0 mL of 64% (w/w) sulfuric acid at 45 °C and stirred for 70 min	TEM micrograph Mean length (L) of the nanostructures was 135.0 ± 12 nm, the mean 269 diameter (D) was 7.2 ± 1.9 nm, and the mean aspect ratio (L/D) was 21.2 ± 2.8	Pereira et al. [54]
Raw cotton linter	Sulfuric acid (60%, w/w) with a Teflon© bar dispersing element, at 45 °C, for 60 min.	TEM micrograph 177 nm long (ranging from 161 to 193), 12 nm wide (ranging from 10 to 13), and have an aspect ratio (L/D) of 19 (ranging from 20 to 24).	Morais et al. [55]
Ushar (*Calotropis procera*) seed fiber	64 wt % sulfuric acid solution with fiber to acid ratio of 1:20 at 50 °C for 75 min with strong agitation	Diameter of 14–24 nm and length of 140–260 nm.	Oun and Rhim [56]
Micro crystalline cellulose	Enzymatic degradation (Clostridium and Coccobacillus–cellulase producing organisms)	AFM micrograph smaller particles 43 ± 13 nm bigger particles 119 ± 9 nm	Satyamurth and Vigneshwaran [57]

**Table 2:** Summary of the nano cellulose-based materials tested in wound healing.

Type of Cellulose	Material Realesed	In Vitro Tests	In Vivo Tests	Molecular Effects	References
PLGA/CNC/CMCS	Cur and ANG	HUVEC cells	Skin full-thickness burn Rat model	Increase of ANG expression, improving wound healing	Mo et al. [70]
Cur/GMs/Col-CNC scaffold	Curcumin	*Escherichia coli, Staphylococcus aureus and Pseudomonas aeruginosa*	Skin full-thickness burn rat model in which the dorsal skin was artificially burned and infected with *Pseudomonas aeruginosa*	in vitro: antimicrobial activity; in vivo: exhibited higher epithelializing rates	Guo et al. [73]
NFC- PEOx-PPOy-PEOx	Octenidine	Vertical diffusion cells and antimicrobial tests		Down regulation of bacterial infection	Alkhatib et al. [74]
NFC	Zinc oxide (ZnO)		Skin burn mice model	Enhanced wound healing and tissue regeneration activity	Khalid et al. [76]
NFC-X		hASC cells		The cells adhere, migrate and proliferate properly	Mertaniemi et al [79]

PLGA/CNC/CMCS = poly(lactic-co-glycolic acid)/cellulose nanocrystals/carboxymethyl chitosan; Cur = curcumin; ANG = angiogenin; Cur/GMs/Col-CNC = Curcumin/gelatin microspheres/collagen/cellulose nanocrystal; NFC- PEOx-PPOy-PEOx = nanofibrillar bacterial cellulose/polyethylene oxide (PEO)/polypropylene oxide (PPO) arranged in a triblock structure; NFC-X = nanofibrillar cellulose with glutaraldehyde cross-links; hASC = human adipose mesenchymal stem cells.

**Table 3:** Summary of the nano cellulose-based materials tested in bone regeneration.

Type of Cellulose	Material Realesed	In Vitro Tests	In Vivo Tests	Molecular Effects	References
SF/CMC		hMSCs		Uniform deposition and nucleation of Ca/P over the surface of the scaffold. The SF/CMC improved osteoblastic differentiation of hMSCs	Singh et al. [81]
Zr-CS-CMC	Cefazolin	Murine OB-6 cell line		Promotion of osteogenic cell proliferation	Gaire et al. [82]
Cotton cellulose	Nano-Ha	HDFCs		Improved HDFCs adherence and proliferation on the scaffold	Ao et al. [84]
CA-P-STMP		Saos-2		Formation of new bone and capillaries	Atila et al. [86]
HPMC-CaP	Calcium	PDECs	Rat bone defect model	In vitro: promotion of the VEGF expression (vasculogenesis); in vivo: increased bone regeneration (osteogenesis) and vessels density	Oliveira et al. [89]

SF/CMC = silk fibroin/Carboxymethylcellulose; hMSCs = human mesenchymal stem cells; Zr-CS-CMC = Zirconium-chitosan- Carboxymethylcellulose; Zr-CS-CMC-P1 = Zirconium-chitosan- Carboxymethylcellulose prepared 1; Zr-CS-CMC-P2 = Zirconium-chitosan- Carboxymethylcellulose prepared 2; nano-Ha = nano-hydroxyapatite; HDFCs = primary human dental follicle cells; CA-P-STMP = Cellulose acetate- Pullulan- trisodium trimetaphosphate; Saos-2 = human Osteogenic Sarcoma Cell Line; HPMC-CaP = Hydroxypropyl)methyl cellulose-calcium phosphate; PDECs = human progenitor-derived endothelial cells.

**Table 4:** Summary of the nano cellulose-based materials tested in cartilage regeneration.

Type of Cellulose	Material Realesed	In Vitro Tests	In Vivo Tests	Molecular Effects	References
NFC/A	hBMSCs and hNC cells		6-weeks-old nude female mice	Cartilage reconstruction and chondrocytes proliferation	Moller et al. [96]
Bacterial NFC bilayer	hNC (in vitro) hNC and MNC (in vivo)	NC cell culture with BCN-bilayer	Nude mice	Chondrogenesis promotion	Martinez et al. [98]

NFC/A = nanofibrillated cellulose/alginate; hBMSCs = human bone marrow–derived stem cells; hNC = human nasal chondrocytes; MNC = bone marrow mononuclear cells.

**Table 5:** Summary of the cellulose-based materials tested in dental applications.

Type of Cellulose	Characteristics	Applications	References
GIC with NFC from eucalyptus pulp	length (L) and diameter (D) of the isolated nanocellulose were determined to be 145 ± 25 nm and 6 ± 1.5 nm, respectively, with an aspect ratio (L/D) of 24.	reinforcing agent in dental restorative material	Silva et al. [99]
Ramie fiber	extracted from the phloem tissue of the plant	reinforcing agent for denture base resin with higher flexural modulus, but weak interfacial adhesion	Xua et al. [103]
Collagen and polylactic acid	Resorbable membrane barriers	disallowing of fast-growing gum tissue from getting into the regenerative site	Fassman, A. et al. [106]
Ethyl cellulose	Non resorbable membrane barriers	disallowing of fast-growing gum tissue from getting into the regenerative site	Fassman, A. et al. [106]
MP containing cellulose	co-polymer of cellulose acetate and nitrocellulose	uniform and enhanced cell migration in bone regeneration	Takata, T. et al. [107]
Polylactic-acid membrane		Less uniform and enhanced cell migration in bone regeneration than MP	Takata, T. et al. [107]
otolith/collagen/bacterial cellulose nanocomposites	Cellulose from bacteria *Gluconacetobacter xylinus*; ultra-fine gel network of cellulose nanofibres (3–8 nm), with nanotolith gels.	Good scaffold for bone and tissue regeneration	Olyveira, G. et al. [108]

MP = Millipore filter; GIC = Glass ionomer cement.

**Table 6:** Summary of the nano cellulose-based materials tested in “other applications”.

Type of Cellulose	Material Realesed	In Vitro Tests	In Vivo Tests	Molecular Effects	References
NFC	Bendamustine	Swelling tests at different pH	Rat model	Good drug loading and release	Bhandari et al. [109]
NFC/RS/P	Methotrexate			In vitro: good delivery and permanence in the colon mucosa	Meneguin et al. [110]
NFC	Metformin	B16F10 cells		Reduction of the migration, invasion and ashesion of B16F10 cells	Nurani et al. [112]
Si-HPMC	hASCs		Rat model	Colonic biological conditions improving in rats with colon damage	Moussa et al. [114]
CNC-PEI	siRNA	C2C12 murine myoblastic cells		Decrease of the cells growth (anti-proliferative)	Ndong Ntoutoume et al. [126]
HEC-PEG	siRNA			Muco-adhesive properties	Furst et al. [128]
CMC/β-C/AA	Acyclovir	Swelling tests at different pH		Basic pH favors the maximal swelling of the CMC/β-C/AA releasing acyclovir in the small intestine	Malik et al. [131]

NFC = Cellulose in form of nanofibers; Si-HPMC = silane-hydroxyl-propylmethyl cellulose; hASC = human adipose mesenchymal stem cells; NFC/RS/P = cellulose nanofibrils/resistant starch/pectin; CMC/β-C/AA = CMC/β-cyclodextrin/acrylic acid; EC-MC = ethyl cellulose-methyl cellulose; CNC-PEI = cellulose nanocrystals-Polyethylenimine; HEC-PEG = hydroxyethyl cellulose-polyethylene glycol; siRNA = small interference RNA.

**Table 7:** Summary of the nano-cellulose-based material tested in clinical trials.

Type of Cellulose	Strategy	Effect	References
EC-MC	Delivery α-mangostin compound	Anti-acne effect	Pan-In et al. [133]
HA-CMC	HA-CMC soaked with Gelfoam	Hearing improvement without significant collateral effects	Ahn et al. [135]
HA-CMC	Artificial tears composed of hyaluronate 0.1% and CMC 0.5%	Improvement of the tear-film in eyes after cataract surgery	Mencucci et al. [136]
HPMC	Delivery oxymetazoline compound	Improvement of mucosal barrier in allergic rhinitis	Valerieva et al. [137]

EC-MC: ethyl cellulose-methyl cellulose; HA-CMC: sodium hyaluronate (HA) and CMC; HPMC: hydroxypropylmethylcellulose.
